# Attitudes towards Peers with Disabilities among Schoolchildren in Physical Education Classes

**DOI:** 10.3390/ijerph20053802

**Published:** 2023-02-21

**Authors:** Jorge Rojo-Ramos, Santiago Gomez-Paniagua, Jose Carmelo Adsuar, Maria Mendoza-Muñoz, Antonio Castillo-Paredes, Angel Denche-Zamorano, Miguel Angel Garcia-Gordillo, Sabina Barrios-Fernandez

**Affiliations:** 1 Physical Activity for Education, Performance and Health, Faculty of Sport Sciences, University of Extremadura, 10003 Cáceres, Spain; 2 BioẼrgon Research Group, University of Extremadura, 10003 Cáceres, Spain; 3 Promoting a Healthy Society Research Group (PHeSO), Faculty of Sport Sciences, University of Extremadura, 10003 Cáceres, Spain; 4 Research Group on Physical and Health Literacy and Health-Related Quality of Life (PHYQOL), Faculty of Sport Sciences, University of Extremadura, 10003 Caceres, Spain; 5 Departamento de Desporto e Saúde, Escola de Saúde e Desenvolvimento Humano, Universidade de Évora, 7004-516 Évora, Portugal; 6 Grupo AFySE, Investigación en Actividad Física y Salud Escolar, Escuela de Pedagogía en Educación Física, Facultad de Educación, Universidad de Las Américas, Santiago 8370040, Chile; 7 Universidad Autónoma de Chile, Talca 3467987, Chile; 8 Occupation, Participation, Sustainability and Quality of Life (Ability Research Group), Nursing and Occupational Therapy College, University of Extremadura, 10003 Cáceres, Spain

**Keywords:** inclusion, attitudes, physical education, disabilities, peers, educational system

## Abstract

All children, including those living with disabilities, have the right to be provided with Inclusive Education (IE) together with appropriate support in scholarly settings. A key factor for educational inclusion is peers’ attitudes towards disabilities, which impact disabled students’ social participation and learning. Physical Education (PE) classes represent an opportunity for students with disabilities to get psychological, social, health, and educational benefits. This study aimed to assess Spanish students’ attitudes regarding their peers with disabilities during PE lessons and to explore possible differences between gender, school location, and age group. The sample consisted of 1437 students from public schools in Extremadura (Spain) from the primary and secondary stages. Participants completed the Attitude towards Students with Disabilities in Physical Education (EAADEF-EP) Questionnaire. The Mann-Whitney U test was carried out to establish differences in scores according to sex, school location, age group, and correlations between age and item scores using the Spearman’s Rho test. The results displayed significant differences in the total and item scores considering sex and centre location, with good reliability values (Cronbach’s alpha = 0.86). The EAADEF-EP Questionnaire has proven to be a quick, easy, and inexpensive tool to assess attitudes. Girls and participants whose school was in a rural setting had better attitudes towards inclusion. This study’s results highlight the importance of carrying out educational actions and programs to improve students’ attitudes towards their peers with disabilities considering the influences of the studied variables.

## 1. Introduction

Inclusive Education (IE) relates to the education that pursues all pupils learning together, whatever their functioning, status, culture, ethnicity, race, sex, sexuality, religion, or language [1,2,3,4]. IE is a multidimensional approach that accepts diversity based on the paradigms of rights and quality of life [5,6], aiming to transform the educational system to enable all learners to live and develop together, providing them with the necessary support to build the basis for a tolerant society [7,8,9,10]. IE promotes the right to education for all students and focuses on partnership, participation, democracy, profit, equal access, quality, equity, and justice. Inclusive schools must create an optimal system that meets every student’s needs and must be prepared to cope with the different demands, so every student should enjoy and be involved in the school’s social relationships, culture, and curriculum [11,12,13]. Therefore, to ensure equal rights for all learners with special educational needs (SEN), IE philosophy and principles have to be adopted [14], with teachers and educational agents being key factors, as they must adapt their daily routines and methodologies [15] and work on their attitudes towards disability, including the possible step from medical and/or paternalistic models (ideas of ‘helping’ students with disabilities) to social and quality of live models [16,17,18]. Inclusion in education involves a focus on student strengths and the provision of support and accommodations to address individual needs. This can take the form of differentiated instruction, the use of technology, and collaboration between teachers, parents, and support staff [4,19]. Additionally, inclusion requires a shift in the culture and attitudes of schools, with a focus on creating welcoming and accepting environments for all students [20]. The IE benefits are well-documented. Students who are included in regular education classrooms have been shown to have higher levels of academic achievement, improved social skills, and a greater sense of belonging in the school community [21,22]. IE also has a positive impact on teachers, as it helps to promote a more positive and supportive learning environment [20,23]. Despite these benefits, there are still many challenges to the full implementation of IE. Teachers often need professional development and support to effectively implement IE strategies in their classrooms. Additionally, there can be resistance from some members of the school community, who may not understand the benefits of IE or may hold negative attitudes towards students with disabilities [20,24].

Attitudes are an important aspect of human behaviour, as they shape our perceptions, thoughts, and behaviours towards people, objects, and events in our environment. Attitudes can be defined as evaluative judgments that can be either positive or negative and influence our actions towards a particular object or person [25,26]. As such, attitudes play a significant role in promoting or hindering IE for students with disabilities. To promote positive attitudes and inclusion in education, it is important to address attitudes and biases through professional development and training for teachers and staff, as well as through education and awareness campaigns for students, families, and communities. In addition, creating inclusive educational environments where students with and without disabilities can learn and interact with each other can also help to promote positive attitudes and inclusion [27,28]. The attitudes of teachers, parents, and peers can greatly impact a student’s experience in an educational setting and their overall academic and social outcomes. Attitudes from educational stakeholders (parents, students, teachers, school directors, politicians, and others) are crucial for successful IE [23]. Research shows that pupils with SEN are less socially engaged than their peers without disabilities [29], with this situation being highly influenced by the type of disability they have [30] and their peers’ attitudes towards their disability [31]. Thus, having pupils with and without disabilities sharing the same classroom will not inevitably lead to more positive attitudes, and adequate mechanisms must be established to move toward IE [32].

Physical Education (PE), Arts, and Music [6] are among the subjects in which inclusive experiences are often implemented, as they offer friendlier contexts [33]. PE has been recognized as an important aspect of a student’s overall education and development: a growing body of research supports the benefits of physical activity for both physical and mental health [34,35]. PE lessons provide a privileged context that can help students with disabilities to feel more like a part of their class or school community, improving their physical health, motor skills, and general well-being [36]. As such, inclusion in PE is critical for promoting social justice and creating an inclusive learning environment for all students according to the National Association for Sport and Physical Education, as students with disabilities are given the opportunity to participate in physical activity alongside their non-disabled peers to the greatest extent possible [37]. PE not only promotes physical health but also helps to break down barriers and promote acceptance and understanding among students of different abilities [27]. Even in a structured and formal educational context, PE lessons can be designated as a facilitator of students’ social development [20]. One of the main factors studied in IE is teacher attitudes [38,39]. Current research reports different points of view, with some studies reporting them as favourable [40] and others showing an imperative need for improvement [28]. These results are based primarily on teachers’ beliefs about students with disabilities; if teachers perceive that these students require more individual attention and support by reducing the tutor’s performance with the rest of the class, their attitudes will be negatively affected [41] since they will think these pupils will disrupt the class by misbehaving or failing to stay on task, resulting in fewer quality lessons for the rest of their pupils [42]. Even though social involvement is crucial for IE, studies reveal that pupils with SEN have fewer social interactions than their classmates without disabilities [14], engage with their classmates less frequently, have fewer friends, and report more feelings of loneliness [43]. One study examining sex and age differences revealed that girls and younger students showed more positive attitudes towards their peers with disabilities. They also observed less accepting attitudes towards peers with behavioural problems versus other problems [44]. Another work found that students in inclusive classes expressed more positive attitudes compared to students in mainstream classes. However, previous contact through joint activities was associated with more positive attitudes, with females being more likely to have positive views than males [45]. Several studies have reported that peers’ attitudes towards their peers with disabilities are not as positive as desirable, emphasising negative attitudes of avoidance and rejection [46]. Contact between students without and students with disabilities is essential, as these encounters can increase positive perceptions which may translate into a greater willingness to interact or play between them [47,48]; having relatives with disabilities was found to be related to more positive perceptions, but a high frequency of interactions happen [45]. However, negative attitudes may contribute to the social isolation of students with disabilities [49], so it is important to promote social inclusion and acceptance during PE lessons, as they are linked to how students without disabilities see and act toward their peers with disabilities [50]. Students in PE classes may be more likely to have negative attitudes towards their peers with disabilities due to a lack of understanding of disability and lack of experience with peers with disabilities. However, the inclusion of peers with disabilities in physical activities and sports can help to improve students’ attitudes towards disability, as contact and cooperation in inclusive sports programs can help to develop more positive attitudes and a greater understanding of disability [51].

This study arises from the need to identify Spanish students’ regarding their peers with disabilities during PE lessons since it is a determining factor for IE. Thus, this research main goal is to evaluate Spanish students’, from Extremadura, attitudes regarding their partners with disabilities in the PE lessons, assessing potential differences between sex, centre location, and age group to draw the current state of this issue and to posteriorly implement pedagogical tools and strategies to improve the positive attitudes toward disability, ensuring proper IE.

## 2. Materials and Methods

### 2.1. Procedure

The Education and Employment Department from the Extremadura Government database was used to select the sample, selecting the centres with primary and secondary stages in which students will be enrolled in PE classes, as it is compulsory. The centers’ management teams were contacted to inform them about the study objectives and to provide them with all the documentation about the study. Informed consent was provided to the centers interested in participating so that they could send it to the parents or guardians of the children who wanted to participate in the study. Once the centres collected them all, an appointment to administer the instruments was established. All participants gave their consent to participate in the study, and a copy of the consent form was provided to the email address they provided when filling in the data. Following different studies that have used similar tools to assess different perceptions in this population [52,53], the surveys (research explanation form, consent, and questionnaire) were elaborated with the Google Forms tool and administered through digital support through electronic devices owned by the research team during the PE class. Electronic surveys were chosen to ease data storage and management because of their higher response rate [54,55]. The average response time was fifteen minutes. Hence, to ensure the items’ understanding, the research team resolved any doubts regarding the instruments.

### 2.2. Measures

The electronic surveys included the following variables:

(a) Sociodemographic Information was obtained using three questions; they answered what their sex was, what the name of the school where they were taught was, and what their age was. For the age groups, three groups were made based on previous literature, considering those under 12 children, those between 12 and 14 adolescents, and those between 15 and 17 older adolescents [56].
(b) The Attitude towards Students with Disabilities in Physical Education Questionnaire (EAADEF-EP) was used to assess students’ attitudes towards disability during PE lessons [57]. The EAADEF-EP is a Spanish-validated tool composed of four items preceded by the sentence “During PE and concerning students with disabilities...”, using a Likert-type scale whose values range from one (strongly disagree) to five (strongly agree). The items were reversed so higher scores revealed a more positive attitude. Furthermore, the EAADEF-EP questionnaire psychometric properties confirmed the unifactorial structure and reports acceptable reliability values through Cronbach’s alpha (>0.79) [58]. The questionnaire was available in Spanish, so no translation or adaptation was necessary.

### 2.3. Participants

Table 1 shows the characteristics of the sample that participated in the study. The sample consisted of 1437 pupils from Public Primary and Secondary stages from Extremadura (Spain) (Table 1). The participants’ mean age was 13.31 years (SD = 3.93). A convenience sampling was used [59].

### 2.4. Statistical Analysis

The Statistical Package for the Social Sciences version 23 was used for the statistical analyses. As variables did not meet the normality assumption, it was decided to use nonparametric statistical tests. The categorical variables are represented by numbers and percentages, and the continuous ones by median and interquartile range. Mann-Whitney U test was employed to analyse the differences between individual items and total EAADEF-EP score according to sex, centre location, and pupils’ age group. Spearman’s Rho test was used to assess the correlations between the items’ results and the student’s ages. The Bonferroni correlation was applied for the *p*-value and set at *p* < 0.016 to establish the significance level. Thus, Cronbach’s alpha was used to calculate the instrument reliability taking as references 0.70 and 0.90 as satisfactory. Furthermore, Cronbach’s alpha and McDonald’s omega were used to assess the reliability of the items and the instrument, considering values above 0.70 as satisfactory [60].

## 3. Results

Table 2 displays the scores obtained in each item according to sex, centre location, and age group. Significant differences were obtained according to sex and centre environment in items 1 “I prefer not to relate to people with disabilities”; 2 “I would avoid doing class work with a person with a disability”; 3 “I would avoid a person with a disability for my team”; and 4 “I would not propose a person with a disability as captain of my team”, with female students from rural centres scoring higher points. Concerning age, no significant differences are obtained for any of the items or the total with the age groups.

Table 3 presents the association between the 4 items of the questionnaire EAADEF-EP and age. The results showed no statistically significant relationship between age and any of the questionnaire items.

Relative to the reliability, Cronbach’s alpha revealed a 0.86 value and internal consistency measures showed values of 0.870 for McDonald’s omega; thus, both were satisfactory.

## 4. Discussion

The main goal of this research is to evaluate Spanish students’ attitudes regarding their peers with disabilities during the PE classes, assessing potential differences between sex, centre location, and age group. In this sense, the main findings of this study showed that girls and students from rural schools obtained better attitudes towards their peers with disabilities in PE classes, with age not being significant in these comparisons.

Students’ sex is a variable that has been strongly related to inclusion in PE across the scientific literature, being the most important predictor of peers’ attitudes, with females having higher positive views about inclusion [61]. Following this line, numerous authors found better attitudes towards the inclusion of students with disabilities in PE lessons on the part of girls compared to boys [62,63,64]. However, some research failed to perceive differences in these attitudes according to sex or considered them too small to generate conclusive principles [65]. These results can be understood from a review of recent years [66], which indicates that girls have different motivations concerning boys in terms of physical activity and sports, being more focused on the enjoyment of the practice and the acquisition of skills so that their vision of participating with students with disabilities is higher. Consequently, most of the literature agrees with our results.

Currently, not much of the literature has compared students’ attitudes towards inclusion by school context. Researchers have focused primarily on the attitudes towards disability of either teachers [67] or university students [68,69]. The few investigations in this area have yielded mixed results. While Parra and Riojas [70] found no difference when considering the location of the school, a study this year identified better attitudes in students from rural schools [71]. Similarly, there is an evident scarcity of studies focused on assessing the differences in these attitudes according to age group. Concerning this, a recent review of studies published between 2012 and 2019 [72] exposes conflicting results in those ages. In this regard, Townsend and Hassal [73] reported more positive values for elementary students compared to their high school peers when it came to including students with cognitive disorders in unified sports. Likewise, Schwab [74] found that the attitudes of primary school children were more positive than those of secondary school children at a general level. In contrast, other studies reported more positive attitudes in older students than juniors [75,76]. Additionally, in line with our results, Loovis and Loovis [77] pointed out that age and the educational stage had no impact on attitudes about inclusion, and Magnusson did not find differences concerning age in children aged 11 to 14 years old [78]. According to the literature, students with and without disabilities benefit from inclusive PE [79]. School inclusion encourages social engagement in various contexts [80] and psycho-emotional well-being [81]. However, the evidence that is now available also shows that exclusion practices are still widely used and may be damaging to kids with disabilities [82]; studies such as this one, which allow us to understand the different mechanisms that govern educational inclusion, are extremely important. Most countries have norms for PE lessons and are a popular way to promote PA throughout the school day worldwide [83]. Therefore, it is essential to comply with WHO recommendations for Physical Activity for children and adolescents [84]. In this line, Kriemler [85] discovered that school-based interventions with many components, instructional, curricular, and environmental, were more effective than those with only instructional or curricular components.

Promoting inclusion in PE classes provides numerous benefits for all students, including those with disabilities. These benefits include: (1) Improved social skills by participating in PE alongside their non-disabled peers; students with disabilities have the opportunity to improve their social skills and develop positive relationships with their peers. This can help to promote a sense of belonging and improve overall well-being. (2) Increased Physical Activity, as all students, including those with disabilities, benefit from increased physical activity. Regular participation in PE can help to improve physical fitness, promote healthy habits, and reduce the risk of chronic health conditions. (3) Enhanced learning, as including students with disabilities in PE classes can enhance learning for all students; by providing accommodations and support to students with disabilities, PE teachers can help to promote a more inclusive and supportive learning environment for all students. (4) Greater understanding: Inclusion in PE can help to promote greater understanding and acceptance of differences among students. This can lead to a more positive and inclusive school culture, where all students feel valued and respected. And (5) improved academic performance as research has shown that regular participation in PE can improve academic performance and attention in the classroom [86,87]. For all these benefits to be realised, it is essential to work on attitudes towards disability in the whole educational community, including, of course, those of classmates.

Therefore, studies such as the present one allow us to know which populations within the student body have better or worse attitudes towards their peers with disabilities, gathering valuable information for both teachers and administrators. This will allow those responsible to have a more specific impact on those students who have negative attitudes towards their peers with disabilities. This can be tackled through a variety of solutions, such as ensuring that education is inclusive and promotes diversity, raising awareness of disability and the rights of people with disabilities, providing positive role models for people with disabilities, and encouraging positive interaction between children and adolescents with and without disabilities. It is important to keep in mind that improving the attitudes of children and adolescents towards peers with disabilities requires a collective effort and an ongoing commitment on the part of the school, family, and community. Therefore, monitoring of these agents influenced children’s attitudes towards inclusion could be an interesting avenue for future research, trying to assess the whole context that may influence children’s and adolescents’ attitudes towards inclusion.

The present study presents several limitations. This research used convenience sampling and was carried out in a single region from Spain, so the results must be assessed cautiously. The sociodemographic factor needs to be improved in further research to better characterize the students and their environmental factors. Finally, online surveys include benefits such as cost savings or ease of data collecting and processing, but they can have drawbacks like the possibility of sample bias, ignorance of the non-respondents’ profiles, and a lower response rate [88]. In the future, studies with information on students with and without disabilities in classes and schools should be added so that comparisons can be established. It would also be interesting to verify awareness-raising programs both at school and within PE classes.

## 5. Conclusions

The present study analysed the possible differences in students’ attitudes towards their partners with disabilities in the PE lessons and found differences between sex and centre location. Specifically, females obtained significantly higher scores for all items of the questionnaire EAADEF-EP. Likewise, participants whose school was located in rural areas also scored significantly higher than participants whose school was located in urban areas. Therefore, females and students from rural schools showed more positive attitudes.

Studies like this could help researchers and teachers to identify attitudes towards the inclusion of students with disabilities in PE. In addition, it could offer PE teachers a general vision of the children and adolescent population according to these attitudes, thus facilitating the detection of attitudes towards the inclusion of certain populations, emphasising those in which the attitudes are less positive, and putting into practice more inclusive methodologies.

## Figures and Tables

**Table 1:** Sample descriptive analysis (*n* = 1437).

Variables	Categories	*n*	%
Sex	Boy	702	48.9
	Girl	735	51.1
Centre location	Urban	664	46.2
	Rural	773	53.8
Age group	6 to 11 years	402	28.0
	12 to 14 years	561	39.0
	15 to 17 years	474	33.0

*n*: number; %: percentage.

**Table 2:** Attitude towards Students with Disabilities in Physical Education Questionnaire scores according to sex, centre location, and age group.

	Sex			Centre Location			Age Group (Years)			
Item	Girls M (SD)	Boys M (SD)	*p*	Rural M (SD)	Urban M (SD)	*p*	6–11 (a) M (SD)	12–14 (b) M (SD)	15–17 (c) M (SD)	*p*
1. I would rather not interact with individuals with disabilities	4.68 (0.66)	4.29 (0.99)	<0.001 *	4.57 (0.82)	4.39 (0.90)	<0.001 *	4.56 (0.74)	4.47 (0.93)	4.45 (0.89)	(ab) 0.638 (ac) 0.280 (bc) 0.525
2. I prefer not to work with a student who has disabilities.	4.54 (0.79)	4.20 (1.07)	<0.001 *	4.51 (0.86)	4.21 (1.02)	<0.001 *	4.33 (0.90)	4.40 (0.99)	4.37 (0.95)	(ab) 0.130 (ac) 0.185 (bc) 0.248
3. For my team, I would avoid students with disabilities.	4.58 (0.70)	4.12 (1.09)	<0.001 *	4.47 (0.85)	4.22 (1.02)	<0.001 *	4.34 (0.95)	4.38 (0.98)	4.33 (0.89)	(ab) 0.201 (ac) 0.586 (bc) 0.058
4. I wouldn’t nominate a person with a disability to be captain for my team.	4.46 (0.92)	3.95 (1.22)	<0.001 *	4.35 (1.01)	4.05 (1.19)	<0.001 *	4.16 (1.17)	4.24 (1.12)	4.22 (1.03)	(ab) 0.220 (ac) 0.864 (bc) 0.275
EAADEF Total	4.56 (0.62)	4.13 (0.93)	<0.001 *	4.47 (0.74)	4.21 (0.87)	<0.001 *	4.56 (0.74)	4.47 (0.93)	4.45 (0.89)	(ab) 0.638 (ac) 0.280 (bc) 0.525

M: mean; SD: Standard deviation; Note: Mann-Whitney U test was significant at * *p* < 0.016.; (a): 6–11-year-old group; (b) 12–14-year-old group; (c) 15–17-year-old group; (ab): *p* for differences between the 6–11-year-old and 12–14-year-old groups; (ac): *p* for differences between the 6–11-year-old and 15–17-year-old groups; (bc): *p* for differences between the 12–14-year-old and 15–17-year-old groups; EEADEF-EP scores (1: “strongly agree”; 2: “agree”; 3: “indifferent”; 4: “disagree”; and 5: “strongly disagree”).

**Table 3:** Correlation coefficients between the Attitude towards Students with Disabilities in Physical Education Questionnaire items and age.

Item	Age ρ (*p*)
1. I would rather not interact with individuals with disabilities	−0.03 (0.174)
2. I prefer not to work with a student who has disabilities.	0.02 (0.289)
3. For my team, I would avoid students with disabilities.	−0.02 (0.320)
4. I wouldn’t nominate a person with a disability to be captain for my team.	−0.01 (0.847)

EEADEF-EP scores (1: “strongly agree”; 2: “agree”; 3: “indifferent”; 4: “disagree”; and 5: “strongly disagree”).

## Data Availability

The datasets used during the current study are available from the corresponding author upon reasonable request.
